# The cytological and electrophysiological effects of silver nanoparticles on neuron-like PC12 cells

**DOI:** 10.1371/journal.pone.0277942

**Published:** 2022-12-13

**Authors:** Zequn Zhang, Chen Meng, Kun Hou, Zhigong Wang, Yan Huang, Xiaoying Lü

**Affiliations:** 1 State Key Laboratory of Bioelectronics, School of Biological Science and Medical Engineering, Southeast University, Nanjing, Jiangsu Province, China; 2 Institute of RF- & OE-ICs, Southeast University, Nanjing, Jiangsu Province, China; 3 Coinnovation Center of Neuroregeneration, Nantong University, Nantong, Jiangsu Province, China; Xiangtan University, CHINA

## Abstract

The aim of this study was to investigate the toxic effects and mechanism of silver nanoparticles (SNPs) on the cytological and electrophysiological properties of rat adrenal pheochromocytoma (PC12) cells. Different concentrations of SNPs (20 nm) were prepared, and the effects of different application durations on the cell viability and electrical excitability of PC12 quasi-neuronal networks were investigated. The effects of 200 μM SNPs on the neurite length, cell membrane potential (CMP) difference, intracellular Ca^2+^ content, mitochondrial membrane potential (MMP) difference, adenosine triphosphate (ATP) content, and reactive oxygen species (ROS) content of networks were then investigated. The results showed that 200 μM SNPs produced grade 1 cytotoxicity at 48 h of interaction, and the other concentrations of SNPs were noncytotoxic. Noncytotoxic 5 μM SNPs significantly increased electrical excitability, and noncytotoxic 100 μM SNPs led to an initial increase followed by a significant decrease in electrical excitability. Cytotoxic SNPs (200 μM) significantly decreased electrical excitability. SNPs (200 μM) led to decreases in neurite length, MMP difference and ATP content and increases in CMP difference and intracellular Ca^2+^ and ROS levels. The results revealed that not only cell viability but also electrophysiological properties should be considered when evaluating nanoparticle-induced neurotoxicity. The SNP-induced cytotoxicity mainly originated from its effects on ATP content, cytoskeletal structure and ROS content. The decrease in electrical excitability was mainly due to the decrease in ATP content. ATP content may thus be an important indicator of both cell viability and electrical excitability in PC12 quasi-neuronal networks.

## Introduction

Due to their excellent antibacterial properties, silver nanoparticles (SNPs) are widely used in a large number of products such as food packaging, air filters, disinfectants, cosmetics, antibacterial gels, trauma dressings and cardiovascular implants [1, 2]. SNPs can enter the circulatory system through oral administration, inhalation, dermal contact and intravenous injection. SNPs smaller than 35 nm in diameter can cross the blood-brain barrier [3] and are subsequently deposited continuously in the brain, causing a variety of pathological responses and diseases [4]. As a result, the toxic effects of SNPs on the nervous system have attracted considerable attention, but the mechanism of toxicity remains unclear.

Unlike normal somatic cells, neurons have different cytological and electrophysiological properties, and their functions are highly dependent on changes in electrophysiological properties. Therefore, we believe that to fully understand the toxicity of SNPs in neurons, both cytological and electrophysiological properties should be comprehensively investigated. However, most of the current studies have focused on the cytological effects of SNPs [5], while very few studies have investigated their electrophysiological effects on neurons [6]. Furthermore, we found no studies that examined or jointly analysed the cytological and electrophysiological effects of SNPs simultaneously.

It is well known that the reception, processing and transmission of information in neuronal networks is the basis of higher brain activities, so studying the electrical excitability of neuronal networks is an effective way to investigate the effects of exogenous factors on brain function in electrophysiological studies. A microelectrode array (MEA) can be used to effectively record the electrical signals of neuronal networks without damaging the neurons [7]. Moreover, measuring electrophysiological characteristics, such as the discharge frequency of neuronal networks cultured on MEAs has been proven to be an effective method for evaluating the toxic effects of exogenous factors (e.g. nanoparticles) on neurons [8]. Our group previously proposed an MEA-based voltage threshold measurement method (VTMM) to quantify the effects of exogenous factors on the electrical excitability of neuronal networks. VTMM is similar to the rheobase measurement with a patch clamp, and both are used to evaluate the electrical excitability by measuring the minimum stimulus that causes excitation [9, 10]. Similar to the rheobase measurement, the lower the voltage threshold (V_Th_) is in VTMM, the higher the electrical excitability is, and vice versa. However, in contrast to the rheobase method, which measures individual neurons, VTMM measures the electrical excitability of a neuronal network. The validity of the VTMM has been verified in our previous studies of hippocampal neuronal networks and hippocampal brain slices [10, 11].

However, using primary cultured neurons has several disadvantages, such as the complexity of extraction, individual variation and potential failure to comply with the 3R (Reduction, Refinement and Replacement) principles for animal protection. In contrast, differentiated rat adrenal pheochromocytoma (PC12) cells are more easily cultured and subcultured. Moreover, PC12 cells not only resemble neurons in terms of cell structure and electrical excitability [12, 13] but are also capable of forming quasi-neuronal networks [14]. Therefore, these cells have been widely used in nanoparticle-induced cytotoxicity and electrophysiology studies [15, 16]. Cui et al. used MEAs and fluorescence microscopy to simultaneously monitor action potentials and dopamine release from PC12 cells [17]. Our group demonstrated that the quasi-neuronal networks had similar electrical excitability changes to those in hippocampal neuronal networks and hippocampal brain slices in response to acetylcholine, ethanol, lidocaine hydrochloride, and temperature changes. Therefore, PC12 cells are recommended as an alternative cell model for studying the cytotoxic and electrical excitability effects of exogenous factors on neurons under certain conditions [18].

The aim of this study was to investigate the toxic effects of SNPs on PC12 cells in terms of changes in both cytological and electrophysiological properties as well as to determine the underlying mechanism of these effects. The effects of SNPs on the viability of PC12 cells and on the electrical excitability of quasi-neuronal networks were first evaluated by the methyl thiazolyl tetrazolium (MTT) method and VTMM respectively. Studies on six aspects of PC12 cells, namely, neurite length, cell membrane potential (CMP) difference, intracellular Ca^2+^ content, mitochondrial membrane potential (MMP) difference, reactive oxygen species (ROS) content and adenosine triphosphate (ATP) content, were subsequently performed using a high content analysis (HCA) system. The correlations among cell viability, electrical excitability and these six indicators after treatment with SNPs were jointly analysed subsequently. Finally, the results above were analysed jointly to elucidate the different mechanisms underlying the SNP-induced changes in cytological and electrophysiological properties.

## Materials and methods

### Preparation and characterization of SNPs

SNPs were prepared by the sodium borohydride reduction of silver nitrate [19]. The morphology of SNPs was assessed using TEM (JEOL JEM-2100, Japan), and size analysis was performed using Image-Pro Plus software v 6.0 (Media Cybernetics, Inc., USA). The concentration of SNPs was measured using an inductive coupled plasma-optical emission spectrometer (Optima 5300DV, Perkin Elmer, USA). Before each experiment, SNPs were diluted to the desired concentration using cell culture medium.

### Cell culture

Differentiated PC12 cells were purchased from Shanghai Cell Bank of Chinese Academy of Sciences. According to the conditions of the culture in the Cell Bank, the cells were adherently cultured in high-glucose DMEM (HyClone, United States) plus 10% fetal bovine serum (Biological Industries, Israel) and 1% (v/v) penicillin-streptomycin (Biological Industries, Israel) [20] and were incubated in a 37 °C, 5% CO_2_ incubator with saturated humidity (Thermo Forma 3111, Thermo Fisher Scientific, United States). In each experiment, cell suspensions were obtained by trypsinization. Cells returned to their normal state 24 h after seeding.

### Evaluation of SNP-induced cytotoxicity in PC12 cells

At the beginning of each experiment, a 96-well plate was filled with 0.1 mg/mL poly-L-lysine solution (Sigma-Aldrich, U.S.) (50 μL/well) to cover all the wells. Then, the plate was placed in an incubator at 37 °C with 5% CO_2_ and saturated humidity for 24 h. Finally, the poly-L-lysine was removed, and the plate was rinsed with sterilized ultra-pure water and dried in a laminar flow cabinet. PC12 cells (100 μL/well) at a concentration of 6 × 10^4^ cells/mL were added to a 96-well plate. After 24 h, the culture medium was aspirated, 5, 25, 50, 100 or 200 μM SNPs dispersed in cell culture medium were added, and PC12 cells were then treated for another 0.5, 1, 12, 24 or 48 h. After rinsing with culture medium, the cytotoxicity of SNPs was evaluated by the MTT method [21]. Cells cultured in medium without SNPs were used as the negative control, and cells cultured in medium containing 0.7% acrylamide were used as the positive control. Six parallel experiments were conducted for each concentration at each time point. According to our previous work [21], toxicity grade 0 means viability rate ∈ [81%, 100%], grade 1 means viability rate ∈ [61%, 80%], grade 2 means viability rate ∈ [41%, 60%], grade 3 means viability rate ∈ [21%, 40%], and grade 4 means viability rate ∈ [0, 20%].

### Preparation of PC12 quasi-neuronal networks on MEAs

At the beginning of each experiment, the MEA (60MEA100/10iR-Ti, Multi Channel Systems MCS GmbH, Germany) was immersed in 75% ethanol for 30 min, dried, and sterilized using ultraviolet light for 8 h. Next, the MEA chamber was filled with sufficient 0.1 mg/mL poly-L-lysine solution to cover all the electrodes. Then, the MEA was placed in an incubator at 37 °C with 5% CO_2_ and saturated humidity for 24 h. Finally, the poly-L-lysine was removed, and the MEA was rinsed with sterilized ultra-pure water and dried in a laminar flow cabinet.

To build PC12 quasi-neuronal networks, 20 μL of differentiated PC12 cells with quasi-neuronal features were seeded onto the surface of the prepared MEA at a cell density of 1×10^6^ cells/mL. Then, the MEA with PC12 cells was placed in an incubator and incubated with the culture medium for approximately 24 h at 37 °C with 5% CO_2_ and saturated humidity. The following experiments could be performed when the PC12 quasi-neuronal networks developed well and covered most of the electrode area on the MEA.

### Measurement of standard V_Th_

**Fig 1:**
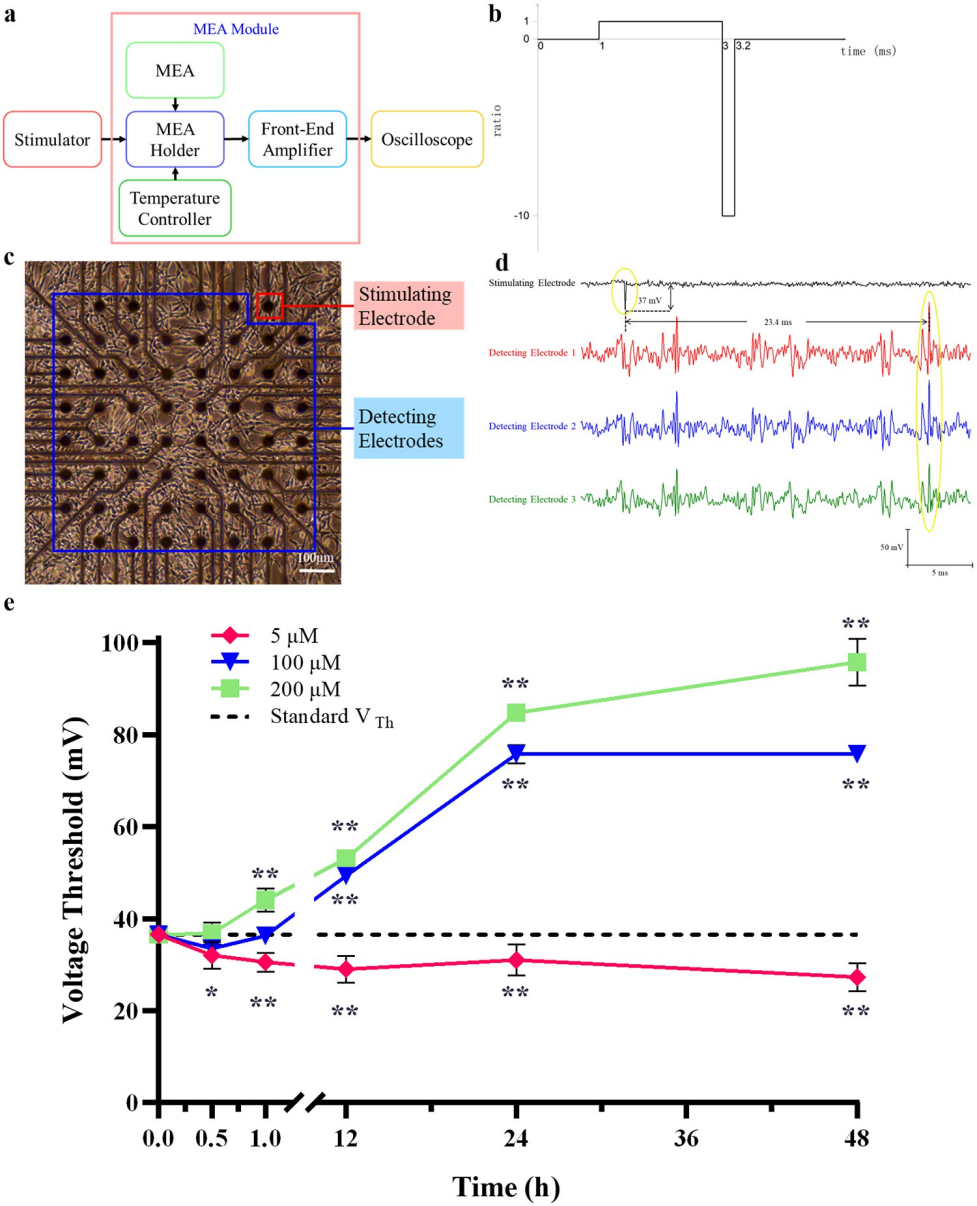
Methods and results of MEA-based VTMM. (**a**) Block diagram showing VTMM system. (**b**) The waveform of voltage stimulation. The stimulation was an asymmetric charge-balanced biphasic pulse at 50 Hz with a positive phase of 2.00 ms and a negative phase of 0.20 ms. (**c**) Image of PC12 quasi-neuronal networks on MEA, with the stimulating electrode in the red box and detecting electrodes in the blue box. The locations of stimulating and detecting electrodes were not fixed in different MEA. Instead, according to the conditions of networks, the electrodes well covered by cells were randomly selected as the stimulating and detecting electrodes, respectively. (**d**) The waveform of the stimulation signal (black) and response signals (red, blue and green) in a normally cultured PC12 quasi-neuronal network (The dataset is shown in S2 Table). (**e**) The V_Th_ in PC12 quasi-neuronal networks treated with 20 nm SNPs at concentrations of 5, 100 and 200 μM for 0.5, 1, 12, 24 and 48 h. * P< 0.05,** P< 0.01. The results are presented as the mean ± s.d. (n = 4).

The experimental procedure and the selection of the voltage stimulation waveform for measuring the normal V_Th_ through VTMM are detailed in our previous paper [10, 11]. In brief, voltage stimulation from one selected stimulating electrode was generated by a voltage pulse generator (Agilent 33220A, USA) and used to trigger responses from the networks (Fig 1a). The stimulation was an asymmetric charge-balanced biphasic pulse at 50 Hz with a positive phase of 2.00 ms and a negative phase of 0.20 ms (Fig 1b). The amplitude of the negative pulse started at 0 mV and increased in steps of 1 mV. An oscilloscope (Agilent 2024A, USA) was used to supervise, recognize, and record the stimulation from the stimulating electrode and the responses from three selected detecting electrodes in real time (Fig 1a). The minimum amplitude of the negative phase of the stimulation pulses that triggered responses from the networks in normal culture environment (with no exogenous factors) was defined as the standard V_Th_. Each MEA was used only once. Four parallel experiments were conducted.

### Measurement of the V_Th_ of networks exposed to SNPs

The original culture medium in the MEA chamber was replaced with fresh test culture medium containing 5, 100 or 200 μM SNPs. After 0.5, 1, 12, 24 or 48 h of incubation, the V_Th_ of the networks was measured as described in Section 2.5. Four parallel experiments were conducted to measure the V_Th_ for each time point under each SNP concentration.

### Measurement of neurite length in cells exposed to SNPs

PC12 cells (100 μL/well) at a concentration of 6 × 10^4^ cells/mL were added to a poly-L-lysine-coated 96-well plate. After 24 h, the culture medium was aspirated, and 200 μM SNPs were added. After PC12 cells were treated for another 0.5, 1, 12, 24 or 48 h, the culture medium was removed, and the cells were fixed with 4% paraformaldehyde and stained with TRITC-conjugated phalloidin and DAPI (Millipore, USA) [22]. The average neurite length was analysed with an HCA system (Array XTI, Thermo Fisher, USA). Cells cultured in medium without SNPs were used as the negative control. Six parallel experiments were conducted at each time point.

### Measurement of CMP differences in cells exposed to SNPs

The CMP difference was monitored by bis-(1,3-dibutylbarbituric acid) trimethine oxonol (DiBAC_4_(3)) (B438, Molecular Probes, United States). DiBAC_4_(3) stock solution (20 mM, dissolved in dimethylsulfoxide) was diluted in Hank’s balanced salt solution (C0218, Beyotime, China) to a working solution (40 μM). PC12 cells were stained with DiBAC_4_(3) working solution in poly-L-lysine-coated 96-well plates (100 μL/well, 1 h) after 0.5, 1, 12, 24 or 48 h of treatment with 200 μM SNPs [23]. The average fluorescence intensity of DiBAC_4_(3) was measured with the HCA system. Cells cultured in medium without SNPs were used as the negative control. Six parallel experiments were conducted at each time point.

### Measurement of intracellular Ca^2+^ content in cells exposed to SNPs

PC12 cells were stained using Fluo-3 AM (S1056, Beyotime, China) in poly-L-lysine-coated 96-well plates after treatment with 200 μM SNPs for 0.5, 1, 12, 24 or 48 h [24]. The average fluorescence intensity of the probes was measured with the HCA system. Cells cultured in medium without SNPs were used as the negative control. Six parallel experiments were conducted at each time point.

### Measurement of MMP differences, ATP content, and ROS content in cells exposed to SNPs

JC-1 (C2006, Beyotime, China) was used to stain PC12 cells after treatment with 200 μM SNPs for 0.5, 1, 12, 24 or 48 h [25]. The average fluorescence intensity of JC-1 aggregates (which produce red fluorescence) and monomers (which produce green fluorescence) was measured with the HCA system. Cells cultured in medium without SNPs were used as the negative control. Six parallel experiments were conducted at each time point.

The ATP content was measured using an ATP assay kit (S0026, Beyotime, China). PC12 cells (1 mL/well) at a concentration of 6×10^4^/mL were cultured in a poly-L-lysine-coated 12-well plate. After 24 h, the culture solution was aspirated, and 1 mL of 200 μM SNPs was added. After 0.5, 1, 12, 24 or 48 h of treatment, the cells were lysed using lysis solution provided by the kit, and the supernatants were collected. The ATP concentrations (C_ATP_) and protein concentrations (C_Protein_) were then detected, and the ATP content was determined as C_ATP_/C_Protein_ and expressed in nmol/mg [26]. Cells cultured in medium without SNPs were used as the negative control. Six parallel experiments were conducted at each time point.

The ROS Assay Kit (S0033S, Beyotime, China) was used to stain PC12 cells in 96-well plates after treatment with 200 μM SNPs for 0.5, 1, 12, 24 or 48 h [26]. The average fluorescence intensity of dichlorofluorescein generated by the oxidation of ROS was measured with the HCA system. Cells cultured in medium without SNPs were used as the negative control. Six parallel experiments were conducted at each time point.

### Data processing and analysis

The Shapiro-Wilk test was employed to test the distribution of the results from parallel groups for normality via SPSS 20.0 software (IBM, Armonk, NY, USA). All experimental data are expressed as the mean ± s.d. ANOVA was followed by the Student-Newman-Keuls (S-N-K) method to test the difference between the groups. The significance levels in the S-N-K method were set to 0.01 and 0.05, respectively. All experiments were repeated at least three times. Pearson correlation analysis was used for the correlation analysis of datasets. The correlation coefficient is abbreviated as “r”; |r| = 0.8 to 1.0 indicates high correlations, |r| = 0.5 to 0.8 indicates moderate correlations, |r| = 0.3 to 0.5 indicates low correlations and |r| = 0 to 0.3 indicates very weak or no correlations [27].

## Results

### Preparation and characterization of SNPs

**Fig 2:**
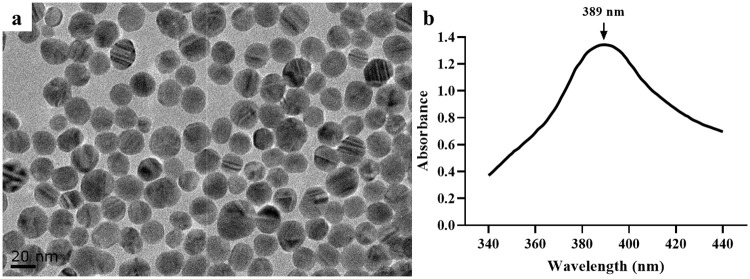
Characterization of SNPs. (**a**) TEM image of SNPs. (**b**) Absorption spectra of SNPs.

The transmission electron microscopy (TEM) image (Fig 2a) and absorption spectra (Fig 2b) of SNPs showed that the SNPs were spherical with an average size of 21.45 ± 2.72 nm and a maximum absorption wavelength of 389 nm (the dataset is shown in S1 Table) [19]. The original concentration of SNPs was 1.4 g/L.

### Evaluation of SNP cytotoxicity in PC12 cells

**Fig 3:**
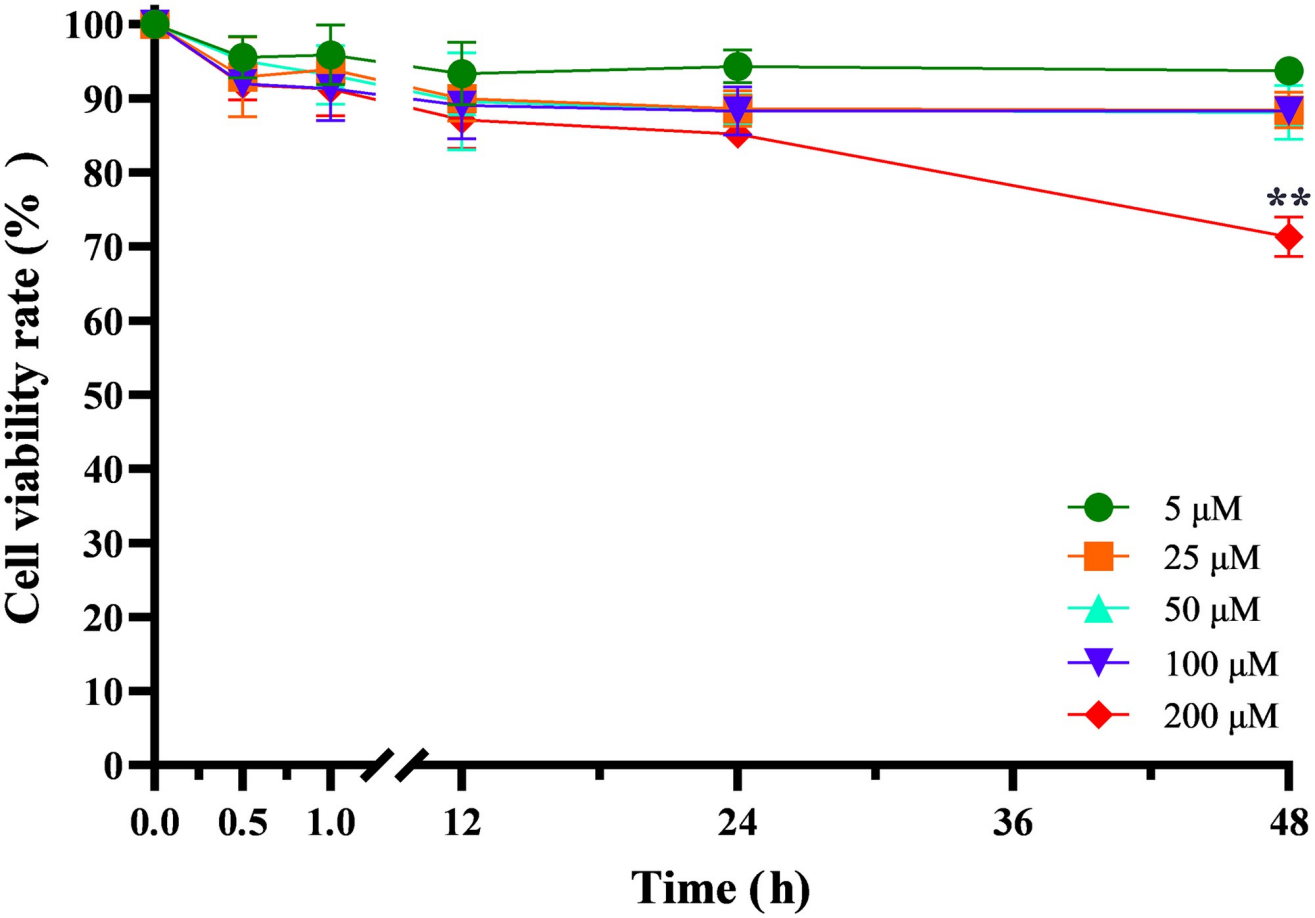
Cell viability rates in PC12 cells. The cell viability rates in PC12 cells treated with 20 nm SNPs at concentrations of 5, 25, 50, 100 and 200 μM for 0.5, 1, 12, 24 and 48 h. The results are presented as the mean ± s.d. (n = 6). Viability rate∈ [81%, 100%] indicates grade 0 cytotoxicity, viability rate∈ [61%, 80%] indicates grade 1 cytotoxicity, viability rate∈ [41%, 60%] indicates grade 2 cytotoxicity, viability rate∈ [21%, 40%] indicates grade 3 cytotoxicity, and viability rate∈ [0, 20%] indicates grade 4 cytotoxicity.

To minimize possible impediments in the MTT assay, the cells were rinsed with culture medium before the experiment [28, 29]. Cell viability rates were normalized to control values. The cell viability rates in PC12 cells treated with different concentrations (5, 25, 50, 100 and 200 μM) of 20 nm SNPs for 0.5, 1, 12, 24 or 48 h are shown in Fig 3. The cell viability rates in all 5 μM SNP-treated groups were higher than 93%. There was no significant difference between the cell viability rates in the 25, 50 and 100 μM SNP-treated groups (P >0.05), and the cell viability rates remained high after 48 h in these groups (88.45±2.41%, 88.10±3.61% and 88.27±0.61%, respectively), indicating grade 0 cytotoxicity. In the 200 μM SNP-treated groups, the cell viability rates were higher than 85% with grade 0 cytotoxicity when the duration of treatment was less than or equal to 24 h. After 48 h, the cell viability rate decreased to 71.29±2.68%, significantly lower than in any other group (P<0.01), indicating grade 1 cytotoxicity.

### Standard V_Th_ in PC12 quasi-neuronal networks

An image of PC12 quasi-neuronal networks on an MEA is shown in Fig 1c, demonstrating that PC12 cells were tightly connected to each other on the MEA. One stimulating electrode and three detecting electrodes were randomly selected among the electrodes covered by cells for experiments. Fig 1d shows the electrical signals of the stimulating and detecting electrodes. As shown in Fig 1d, 23.4 ms after stimulation (negative pulse amplitude of 37 mV) was applied to one normally cultured PC12 quasi-neuronal network, action potentials (5 × rms noise [30]) were recorded at each of the three detecting electrodes. Therefore, the standard V_Th_ in this specific PC12 quasi-neuronal network (shown in Fig 1) was 37 mV.

According to our previous results, the minimum amplitude of the stimulation pulses that triggered responses from the networks in a normal culture environment was defined as the standard V_Th_. The V_Th_ in networks of the same kind of cells is a fixed value as the test environment remains unchanged [10, 11]. The standard V_Th_ in PC12 quasi-neuronal networks measured in this study was 36.50 ± 0.58 mV, which was not significantly different (P > 0.05) from our previous result (36 ± 2.37 mV) [18].

### V_Th_ in PC12 quasi-neuronal networks exposed to SNPs

The V_Th_ in PC12 quasi-neuronal networks treated with 20 nm SNPs at concentrations of 5, 100 or 200 μM for 0.5, 1, 12, 24 or 48 h are shown in Fig 1e.

After treatment with 5 μM SNPs, the V_Th_ in PC12 quasi-neuronal networks was significantly lower than the standard V_Th_ at all five time points (P<0.05), with a minimum value of 27.25 ± 1.89 mV at 48 h, which suggests that 5 μM SNPs led to a significant increase in the electrical excitability of PC12 quasi-neuronal networks.

After treatment with 100 μM SNPs, the V_Th_ in the networks was lower than the standard V_Th_ at 0.5 and 1 h and significantly higher than the standard V_Th_ at 12 h and beyond (P<0.01). This result suggests that 100 μM SNPs led to an increase followed by a significant decrease in the electrical excitability of PC12 quasi-neuronal networks.

After treatment with 200 μM SNPs, the V_Th_ in the networks was higher than the standard V_Th_ at each time point and was significantly higher than the standard V_Th_ at 1 h and beyond (P<0.01). This result suggests that 200 μM SNPs caused a significant decrease in the electrical excitability of PC12 quasi-neuronal networks after 1 h of treatment.

### Neurite length in cells exposed to SNPs

**Fig 4:**
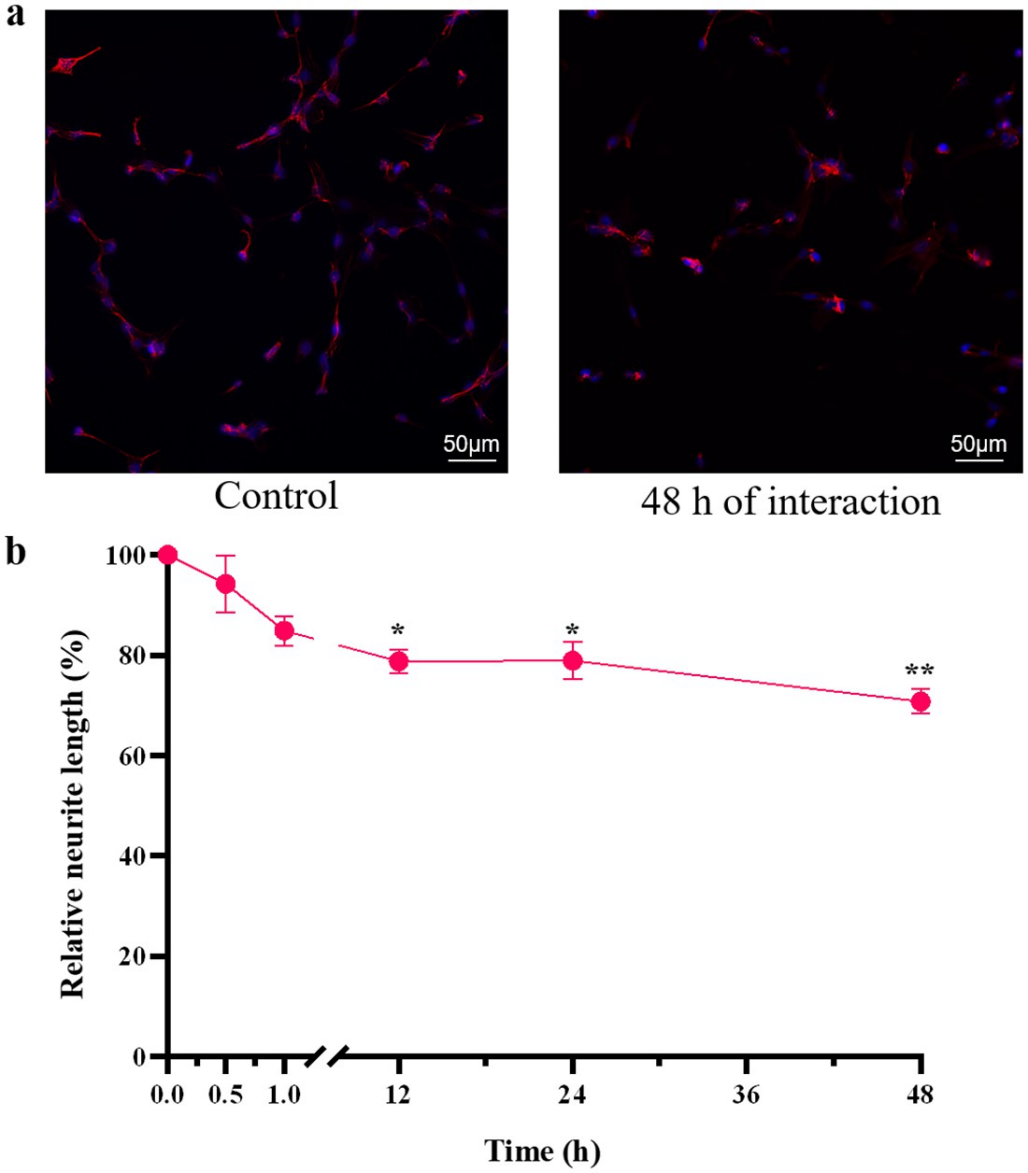
Neurite length in PC12 cells. (**a**) The fluorescent images of PC12 cells stained with TRITC-conjugated phalloidin and DAPI. The images were gray-scale images with pseudo color, where the neurites were red, and the nuclei were blue. (**b**) The neurite length in PC12 cells treated with 20 nm 200 μM SNPs for 0.5, 1, 12, 24 and 48 h. * P< 0.05, ** P< 0.01. The results are presented as the mean ± s.d. (n = 6).

The neurite length data were normalized to control values. The lengths of neurites in PC12 cells treated with 20 nm 200 μM SNPs for 0.5, 1, 12, 24 or 48 h are shown in Fig 4. After treatment with 200 μM SNPs, the neurite length decreased gradually and was significantly (P<0.05) lower than that in the control group after 12 h.

### CMP difference in cells exposed to SNPs

**Fig 5:**
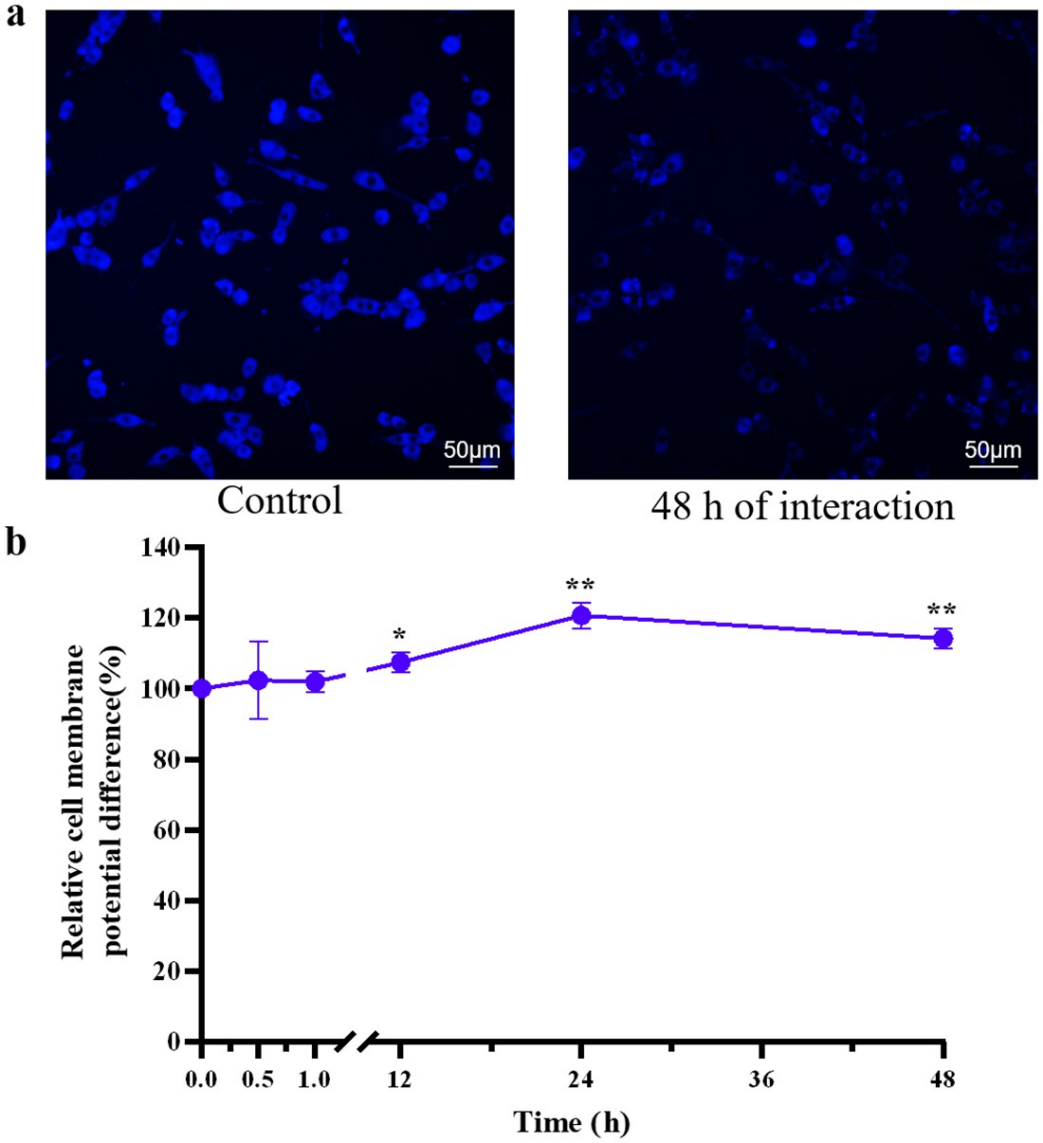
CMP difference in PC12 cells. (**a**) The fluorescent images of PC12 cells stained with DiBAC_4_(3). The images were gray-scale images with pseudo color, where the weaker the blue fluorescence, the higher the CMP difference. (**b**) The CMP differences in PC12 cells treated with 20 nm 200 μM SNPs for 0.5, 1, 12, 24 and 48 h. *P< 0.05,**P< 0.01. The results are presented as the mean ± s.d. (n = 6).

Data regarding the CMP difference were normalized to control values. The CMP differences in PC12 cells treated with 20 nm 200 μM SNPs for 0.5, 1, 12, 24 or 48 h are shown in Fig 5. The CMP differences in each SNP-treated group were higher than those in the control group. Within 0–24 h, noncytotoxic concentrations of SNPs induced a sustained increase in CMP difference. A significantly lower CMP difference was observed at 48 h than 24 h (P<0.05). However, at 48 h, the CMP difference was still significantly higher than that in the control group (P<0.01), and the V_Th_ was still significantly higher than the standard V_Th_ (P<0.01) (Fig 1e), which suggests that the electrical excitability of these networks was still lower than normal.

### Intracellular Ca^2+^ content in cells exposed to SNPs

**Fig 6:**
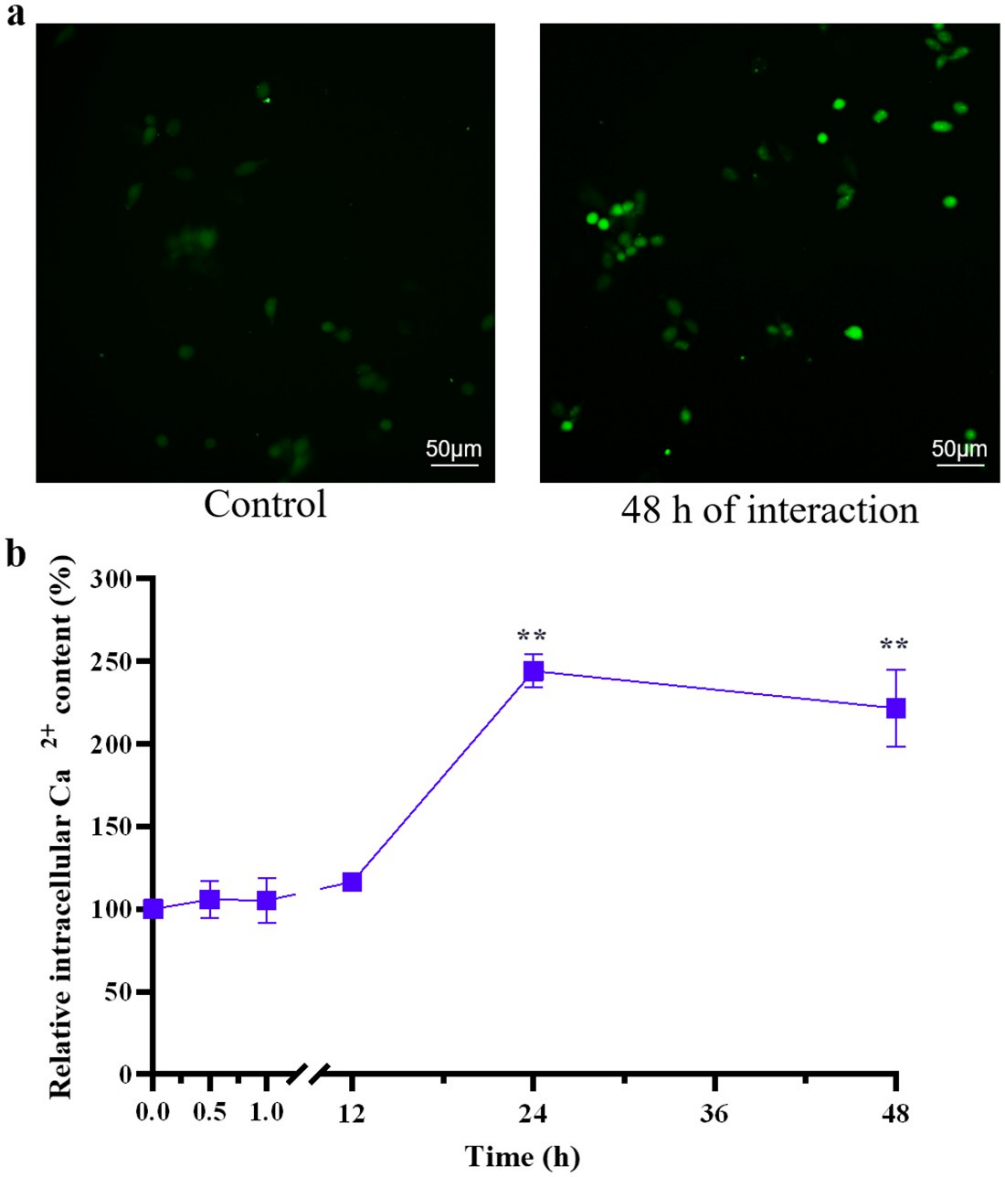
Intracellular Ca^2+^ content in PC12 cells. (**a**) The fluorescent images of PC12 cells stained with Fluo-3 AM. The images were gray-scale images with pseudo color, where the brighter the green fluorescence, the higher the Ca^2+^ content. (**b**) The intracellular Ca^2+^ content in PC12 cells treated with 20 nm 200 μM SNPs for 0.5, 1, 12, 24 and 48 h. ** P< 0.01. The results are presented as the mean ± s.d. (n = 6).

Intracellular Ca^2+^ content data were normalized to control values. The intracellular Ca^2+^ contents in PC12 cells treated with 20 nm 200 μM SNPs for 0.5, 1, 12, 24 or 48 h are shown in Fig 6. The intracellular Ca^2+^ contents in all SNP-treated groups were higher than those in the control group and were significantly higher at 24 h (243.96%) and 48 h (221.50%) (P<0.01). Moreover, the intracellular Ca^2+^ content at 48 h was significantly lower (P<0.05) than that at 24 h.

### MMP difference, ATP content and ROS content in cells exposed to SNPs

**Fig 7:**
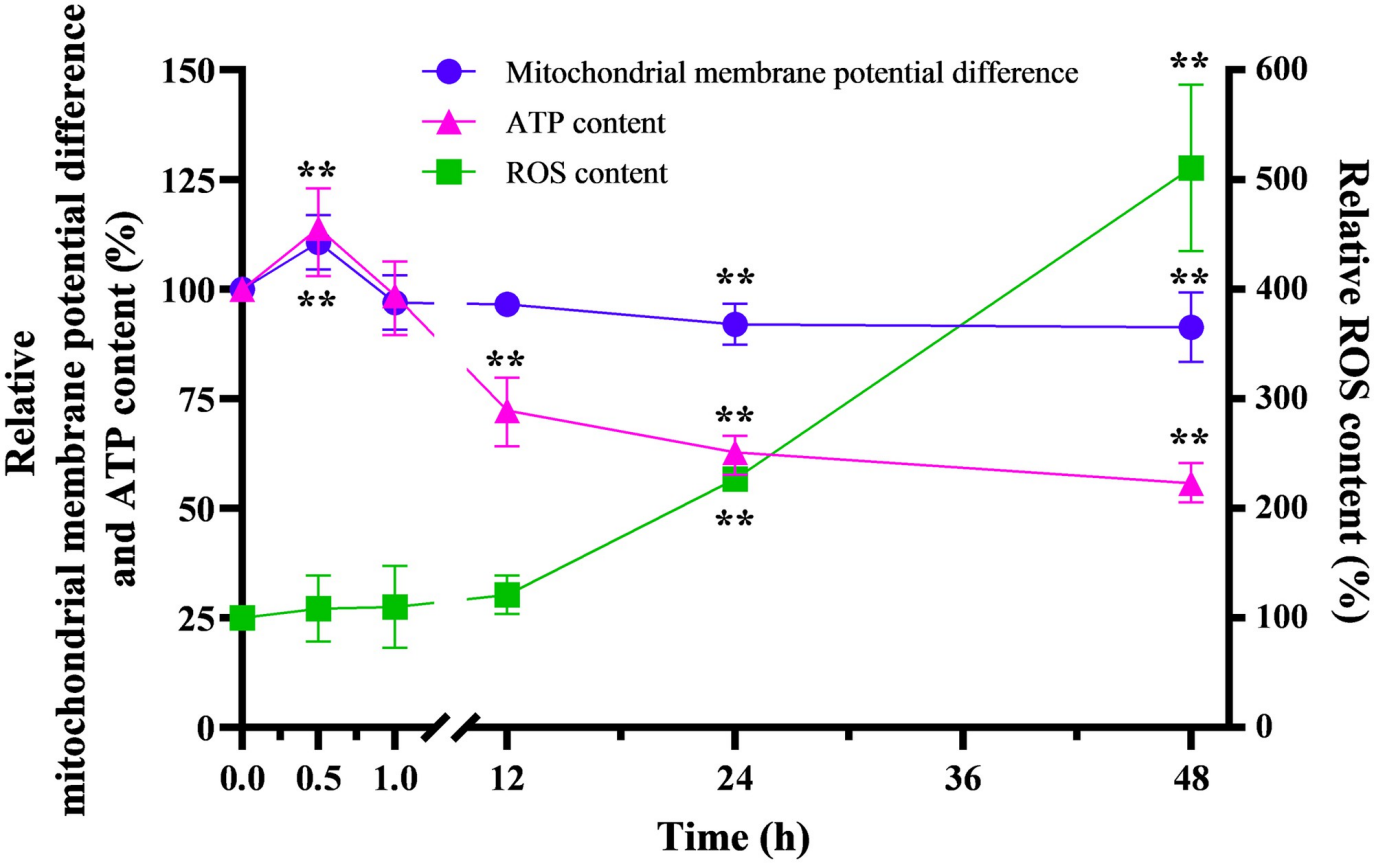
MMP difference, ATP content and ROS content in PC12 cells. MMP difference, ATP content and ROS content in PC12 cells treated with 20 nm 200 μM SNPs for 0.5, 1, 12, 24 and 48 h. *P< 0.05,**P< 0.01. The results are presented as the mean ± s.d. (n = 6).

Data regarding the MMP difference, ATP content and ROS content were normalized to control values. The MMP difference, ATP content and ROS content in PC12 cells treated with 200 μM SNPs for 0.5, 1, 12, 24 or 48 h are shown in Fig 7. The MMP difference and ATP content in cells exposed to SNPs were significantly higher (P<0.01) than those in the control groups at 0.5 h of treatment. The MMP difference and ATP content continuously decreased starting at 1 h and were significantly lower than those in the control group at 24 h and 48 h and at 12 h, 24 h and 48 h, respectively (P<0.01). The ROS content in cells exposed to SNPs continuously increased and was significantly higher than that in the control group at 24 and 48 h (P<0.01).

### Conjoint analysis between cytological indicators, cell viability and V_Th_

Correlation analysis of cell viability and V_Th_ in cells exposed to SNPs revealed a high correlation (r = -0.95). Thus, to investigate the molecular mechanisms underlying cell viability and V_Th_ variation, the results of cell viability and V_Th_ were analysed conjointly with the results of the above six cytological indicators, and Pearson correlation coefficients were calculated for the relationships between cell viability, V_Th_ and each indicator (Table 1).

**Table 1:** Pearson correlation coefficients (r) between each cytological indicator, cell viability and V_Th_.

	V_Th_	ATP content	Neurite length	ROS content	Intracellular Ca^2+^ content	MMP difference	CMP difference
Cell viability	**-0.95**	**0.95**	**0.93**	**-0.90**	**-0.83**	0.77	-0.79
V_Th_		**-0.92**	**-0.85**	**0.87**	**0.96**	-0.79	**0.91**

|r| = 0.8 to 1.0 indicates high correlation, |r| = 0.5 to 0.8 indicates moderate correlation, |r| = 0.3 to 0.5 indicates low correlation and |r| = 0 to 0.3 indicates very weak or no correlation.

Both PC12 cell viability and V_Th_ were highly correlated with ATP content, neurite length, ROS content and intracellular Ca^2+^ content (|r|>0.8), and both were moderately correlated with MMP difference (r = 0.77 and -0.79). Additionally, V_Th_ was highly correlated with CMP difference (r = 0.91), while cell viability was only moderately correlated with CMP difference (r = -0.79).

## Discussion

SNPs smaller than 35 nm are able to cross the blood-brain barrier [3]. The size of SNPs commonly used in clinical practice is generally ~20 nm, and SNPs of this size have been used in neurotoxicity studies by several research groups [31, 32]. Therefore, 20 nm SNPs were selected in this study to investigate their toxic effects.

In the current study, the MTT results showed that 200 μM SNPs produced grade 1 cytotoxicity at 48 h of interaction, and the other concentrations of SNPs were noncytotoxic. The size of the nanoparticles is one of the factors affecting their cytotoxicity, and it has been found that the cytotoxic effect of SNPs with small sizes is greater than that of SNPs with large sizes. Akter et al. found that the survival of PC12 cells was reduced to 23% at 48 h of the interaction of 10 nm SNPs at a concentration of 3 ppm [33], and the cytotoxic effect of 10 nm SNPs in this study was higher than that of 20 nm SNPs in this paper. In our previous study, after 72 h of interaction on human dermal fibroblasts, 5 nm SNPs led to a cell survival rate of less than 20%, while 20 nm SNPs only led to a cell survival rate of 70.44% [34]. Mishra et al. found that the toxicity of 10 nm SNPs in human liver-derived hepatoma cells was higher than that of 50 and 100 nm SNPs [35].

To investigate the effects of noncytotoxic and cytotoxic concentrations of SNPs on PC12 quasi-neuronal networks and to compare our current findings with our previous work [36], three concentrations of SNPs (5, 100 and 200 μM) were selected for VTMM experiments.

Fig 1e suggests that 5 μM SNPs led to a significant increase in the electrical excitability of PC12 quasi-neuronal networks, which was consistent with our previous work [36]. In addition, 100 μM SNPs led to an increase followed by a significant decrease in the electrical excitability of the networks. This also suggests that 200 μM SNPs caused a significant decrease in the electrical excitability of the networks after 1 h of treatment.

When comparing Figs 3 and 1e, it was evident that the noncytotoxic 5 μM SNPs led to a significant decrease (P<0.05) in the V_Th_ in PC12 quasi-neuronal networks at 0.5 h, while noncytotoxic 100 μM SNPs led to a significant increase (P<0.01) in the V_Th_ in the networks at 12 h. This result indicates that SNPs were still able to alter the electrical excitability of PC12 quasi-neuronal networks at noncytotoxic concentrations. Grade 1 cytotoxicity appeared after 48 h of treatment with 200 μM SNPs, yet the V_Th_ was significantly (P<0.01) higher than the standard V_Th_ after only 1 h, which indicated a reduction in the electrical excitability of PC12 quasi-neuronal networks. Taken together, these results show that the SNP-induced changes in the electrophysiological properties of PC12 cells appeared before the changes in cell viability, suggesting that using cell viability alone to evaluate nanoparticle-induced neurotoxicity is partial. Therefore, not only cell viability but also electrophysiological properties should be considered when evaluating nanoparticle-induced neurotoxicity.

To further investigate the mechanisms of SNP-induced cytotoxicity and changes in electrical excitability, changes in six aspects of PC12 cells, namely, neurite length, CMP difference, intracellular Ca^2+^ content, MMP difference, ROS content and ATP content were studied under the effect of 200 μM cytotoxic SNPs.

On the one hand, the presence of neurites is a prerequisite for forming synapses, which are the basis of signaling between neurons. In addition, a relatively high density of voltage-gated sodium channels, which play an important role in the production and conduction of neural signals, is distributed along the axon initiation segment of neurons [37]. Decreased neurite length might shorten the axon initiation segment, therefore leading to a decrease in the number of sodium channels, which in turn would affect neurotransmitter release and reduce the electrical excitability of neuronal networks [11]. On the other hand, decreased neurite length corresponds to cell damage, particularly cytoskeletal damage [38]. Thus, the SNP-induced decrease in neurite length (Fig 4) suggests that SNPs caused cytoskeletal damage and that this decrease might affect signaling between neuron-like PC12 cells.

CMP is one of the most important indicators of cell survival, as a decrease in CMP difference is usually accompanied by toxic effects, apoptosis and necrosis [39]. Additionally, the CMP difference directly influences the resting potential and polarization of neurons, rendering it a pivotal factor affecting the electrical excitability of neurons [40]. Within 0–24 h, noncytotoxic concentrations of SNPs induced a sustained increase in CMP difference (Fig 5), which indicated cell hyperpolarization [41], thereby contributing to reduced electrical excitability (Fig 1e). A significantly lower CMP difference was observed at 48 h than 24 h (P<0.05). This result might have been due to the cytotoxicity of SNPs at 48 h, as cell death is usually accompanied by a decrease in CMP difference [42].

The intracellular Ca^2+^ contents in all SNP-treated groups were higher than those in the control group (Fig 6). These results are consistent with the finding of Ziemińska et al. that 75 μg/mL SNPs that were 5–35 nm in size could lead to elevated Ca^2+^ levels in cerebellar granule cells due to the overactivation of NMDA receptors [43]. Moreover, the intracellular Ca^2+^ content at 48 h was significantly lower (P<0.05) than that at 24 h. This finding might be attributed to the significant increase in intracellular Ca^2+^ content at 24 h, which initiated negative feedback regulation, followed by the inactivation of NMDA receptors [44], resulting in a decrease in intracellular Ca^2+^ content at 48 h. Overloading intracellular Ca^2+^ leads to mitochondrial and cytoskeletal damage and even apoptosis [45]. Moreover, Ca^2+^ is an important intracellular signaling molecule that can control neurotransmitter release, regulate the expression of various proteins and influence the excitability of neurons [46]. In the current study, 200 μM SNPs applied to PC12 cells for 48 h were found to simultaneously induce grade 1 cytotoxicity (Fig 3), significantly increase V_Th_ (Fig 1e) and significantly increase intracellular Ca^2+^ content (Fig 6).

The mitochondrion is an important organelle in the cell, as it is the site of energy production and one of the important targets of nanoparticles to induce toxicity [2]. The MMP difference is an important indicator of mitochondrial function. A decreased MMP difference implies mitochondrial damage and is one of the early signs of apoptosis [47]. Additionally, impaired mitochondria can decrease ATP (an important cellular energy source) content, which can lead to cellular energy deficiency [48]. A previous study by our group revealed that the ATP content in human dermal fibroblasts decreased under the effect of SNPs [49]. Moreover, electrons escaping from a damaged mitochondrial electron transport chain might directly react with substances such as oxygen and generate ROS [50]. Damage to mitochondria therefore leads to increases in ROS content, and excessive ROS levels might contribute to further cellular damage (e.g., DNA, protein, and synaptic damage) [51]. Thus, ROS content is one of the most important indicators of cellular damage and the effect of nanoparticles on cells. The results displayed in Fig 7 indicate that treatment with SNPs for 24 and 48 h caused mitochondrial damage which reduced the MMP difference, contributing to decreases in ATP content and increases in ROS content. Additionally, the MMP difference and ATP content in cells exposed to SNPs were significantly higher (P<0.01) than those in the control groups at 0.5 h of treatment, which could have been caused by the metabolic adaptation to the presence of SNPs. Metabolic adaptation might increase the MMP difference and enhance the tricarboxylic acid cycle of mitochondria, resulting in increased ATP production [52].

The three indicators most highly correlated with cell viability in Table 1 were ATP content (r = 0.95), neurite length (r = 0.93), and ROS content (r = -0.90). SNPs have been found to simultaneously decrease ATP content and suppress neurite growth in human embryonic stem cell-derived neurons [53] and to decrease MMP difference, increase ROS content and decrease viability in A549 cells [54]. The current study demonstrated that ATP content, neurite length and ROS content were important indicators of cellular damage caused by nanoparticles. The main cause of SNP-induced cytotoxicity was the detrimental effects on cellular energy supply, cytoskeletal integrity and ROS content.

Elevated intracellular Ca^2+^ content has been found to contribute to reduced ATP synthesis [45] and cellular energy deficiencies, which in turn open ATP-sensitive potassium (K_ATP_) channels on the cell membrane, resulting in increases in CMP difference and hyperpolarization [41]. This study showed that SNPs increased intracellular Ca^2+^ content (Fig 6), decreased ATP content (Fig 4), increased CMP difference (Fig 5) and increased V_Th_ (Fig 1e) in PC12 cells. Furthermore, the three indicators most highly correlated with V_Th_ (displayed in Table 1) were intracellular Ca^2+^ content (r = 0.96), ATP content (r = -0.92) and CMP difference (r = 0.91). The above results suggest that the SNP-induced decrease in electrical excitability may be explained by a decrease in ATP content due to an increase in intracellular Ca^2+^ content, which led to cellular energy deficiency that opened K_ATP_ channels on the cell membrane, resulting in an increase in CMP difference and hyperpolarization.

Additionally, ATP content was the only cytological indicator that correlated with both cell viability and V_Th_, with correlation coefficients above 0.9. This result indicates that the ATP content was the main cytological indicator that affected both cytotoxicity and electrical excitability in the presence of SNPs and illustrates the importance of energy supply for the maintenance of neuronal cell structure and function.

**Fig 8:**
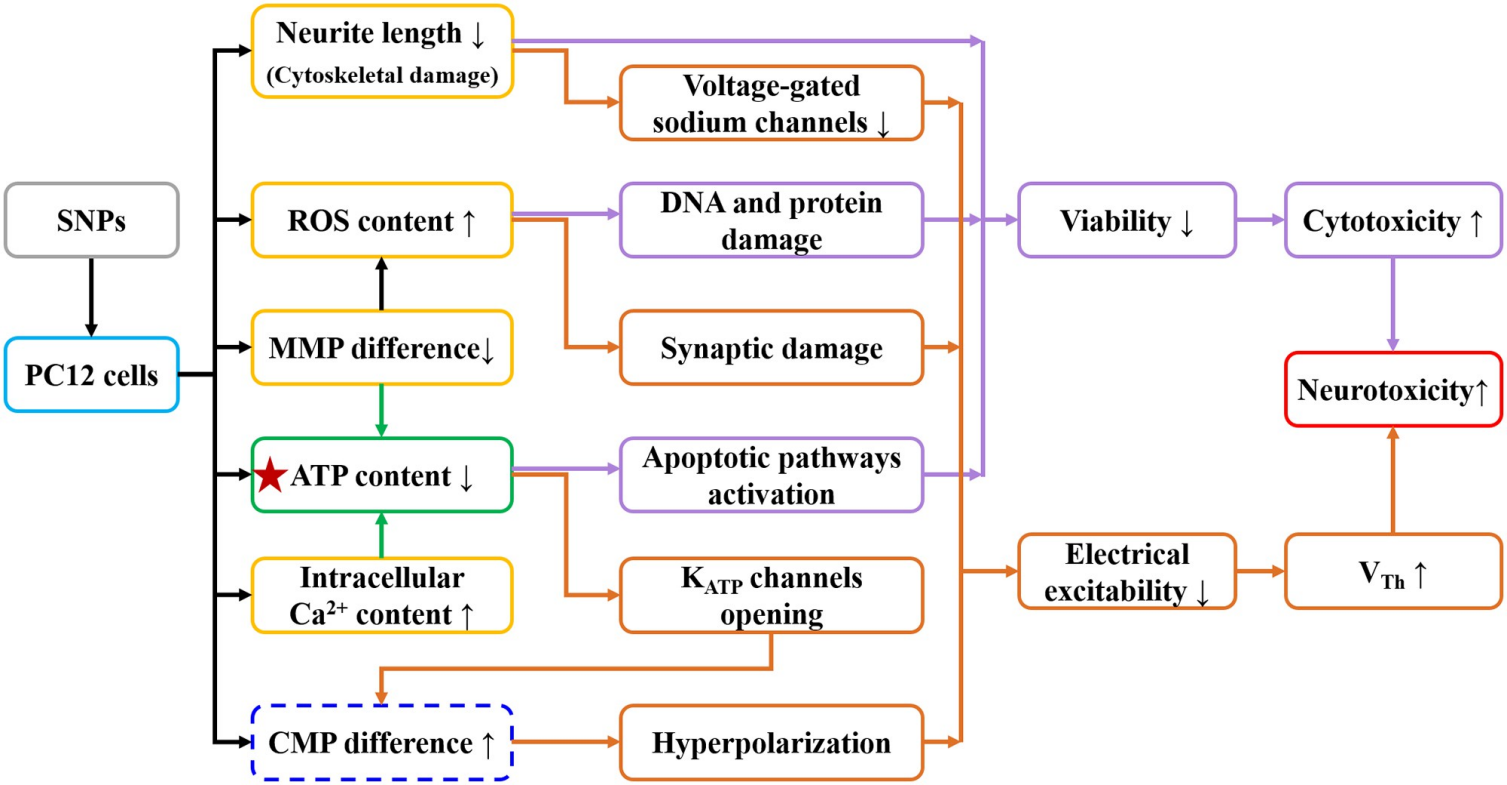
Diagram of possible mechanisms for the changes in cytotoxicity and electrical excitability of PC12 cells caused by SNPs.

Possible mechanisms for the SNP-induced changes in cytotoxicity and electrical excitability of PC12 cells are summarized in Fig 8. SNPs decreased neurite length in PC12 cells, suggesting that SNPs caused cytoskeletal damage [38] and cytotoxicity. In addition, decreased neurite length might reduce voltage-gated sodium channels and diminish the electrical excitability of PC12 quasi-neuronal networks [11]. The SNP-induced decrease in MMP difference increased ROS content, which might damage biomolecules such as DNA and proteins, leading to cell death [51]. Moreover, increased ROS content might impair synaptic structures, which could affect intercellular signaling [55] and reduce the electrical excitability of PC12 quasi-neuronal networks. Both the decrease in MMP difference and the increase in intracellular Ca^2+^ content could lead to a decrease in ATP content. Decreased ATP content could lead to cellular energy deficiency, which could activate apoptotic pathways [56], causing a decrease in cell viability, and could open K_ATP_ channels [41], causing an increase in CMP difference and hyperpolarization, eventually resulting in reduced electrical excitability.

Many receptors of PC12 cells are identical to those of rat primary neurons. Compared to primary neurons, PC12 cells can more quickly form a cellular network on the MEA that can transmit electrical signals with a simpler culture process and a single cell type [18]. Therefore, using PC12 cells as a simple neuronal network model to study the mechanism of the toxic effects of SNPs on neurons can effectively avoid the interference caused by the complex cellular composition of the primary neuronal networks. However, the simplicity of PC12 cells also limits their ability to mimic normal neuronal networks fully. Human iPSC-derived neurons-based 3D brain organoids containing a variety of neurons and glial cells possess a more complete organization and are more similar to the human central nervous system [57]. The toxic effects of silver nanoparticles [58] and zinc oxide nanoparticles [59] have been investigated using brain organoids. Researchers also use MEA [60] and whole-cell patch-clamp [61] to record brain organoids’ electrophysiological response to external stimulation. According to this study, it is suggested to combine cytological and electrophysiological methods to comprehensively evaluate nanoparticle-induced neurotoxicity when using brain organoids as an experimental model.

## Conclusion

In this study, the effects of SNPs on the viability and electrical excitability of PC12 cells were simultaneously investigated using the MTT method and VTMM. SNPs were found to alter the electrical excitability of PC12 quasi-neuronal networks at noncytotoxic concentrations. At the cytotoxic concentration, SNPs altered electrical excitability before they altered cell viability, suggesting that using only cell viability to evaluate nanoparticle-induced neurotoxicity is partial. Therefore, not only cell viability but also electrophysiological properties should be considered when evaluating nanoparticle-induced neurotoxicity. Furthermore, the effects of SNPs on the six cytological indicators of PC12 cells were investigated and analysed jointly with cell viability and electrical excitability, revealing that the main reason for SNP-induced cytotoxicity was the detrimental effects on cellular energy supply, cytoskeletal integrity and ROS content. The SNP-induced decrease in electrical excitability could be explained by a decrease in ATP content caused by mitochondrial damage and increased intracellular Ca^2+^ content. A decrease in ATP content could lead to cellular energy deficiency that opened K_ATP_ channels on the cell membrane, resulting in an increase in the CMP difference and hyperpolarization. ATP content was the main cytological indicator of both cytotoxicity and electrical excitability in the presence of SNPs. By jointly studying the cytological and electrophysiological effect of SNPs on PC12 cells, this study provides a new direction for evaluating the neurotoxicity induced by nanoparticles.

## Supporting information

S1 Table Dataset of Fig 2b. Absorption spectra of SNPs.
(XLSX)

S2 Table Dataset of Fig 1d. The stimulation signal (Channel 1) and response signals (Channel 2, 3 and 4) in a normally cultured PC12 quasi-neuronal network.
(XLSX)
